# Pleiotropic Effects of Biguanides on Mitochondrial Reactive Oxygen Species Production

**DOI:** 10.1155/2017/7038603

**Published:** 2017-08-09

**Authors:** Alena Pecinova, Zdenek Drahota, Jana Kovalcikova, Nikola Kovarova, Petr Pecina, Lukas Alan, Michal Zima, Josef Houstek, Tomas Mracek

**Affiliations:** Institute of Physiology of the Czech Academy of Sciences, Vídeňská, 1083 Prague, Czech Republic

## Abstract

Metformin is widely prescribed as a first-choice antihyperglycemic drug for treatment of type 2 diabetes mellitus, and recent epidemiological studies showed its utility also in cancer therapy. Although it is in use since the 1970s, its molecular target, either for antihyperglycemic or antineoplastic action, remains elusive. However, the body of the research on metformin effect oscillates around mitochondrial metabolism, including the function of oxidative phosphorylation (OXPHOS) apparatus. In this study, we focused on direct inhibitory mechanism of biguanides (metformin and phenformin) on OXPHOS complexes and its functional impact, using the model of isolated brown adipose tissue mitochondria. We demonstrate that biguanides nonspecifically target the activities of all respiratory chain dehydrogenases (mitochondrial NADH, succinate, and glycerophosphate dehydrogenases), but only at very high concentrations (10^−2^–10^−1^ M) that highly exceed cellular concentrations observed during the treatment. In addition, these concentrations of biguanides also trigger burst of reactive oxygen species production which, in combination with pleiotropic OXPHOS inhibition, can be toxic for the organism. We conclude that the beneficial effect of biguanides should probably be associated with subtler mechanism, different from the generalized inhibition of the respiratory chain.

## 1. Introduction

Metformin (dimethyl biguanide) is the most widely used frontline drug for treatment of type II diabetes mellitus [1, 2]. At the whole-body level, it effectively decreases blood glucose and insulin levels during hyperglycemia [3]. Several possible mechanisms of its action have been suggested, including inhibition of adenylate cyclase [4], interference with mitochondrial dynamics [5], alterations in gut microbiota composition [6], or inhibition of mitochondrial respiratory chain [7, 8]. Some of the proposed mechanisms oscillate around AMP-activated protein kinase (AMPK) activation, which in itself was also suggested as a direct target for biguanides [5, 9]. However, precise molecular mechanism of its action remains questionable [10, 11].

Metformin utility was also explored in the model of heart failure where epidemiologic evidence suggests its protective effect [12, 13]. Nevertheless, at the molecular level, a direct effect on mitochondria is observed in some cases [14], but not in others [15].

In addition to its antihyperglycemic effect, a broad range of epidemiologic studies showed that chronic metformin treatment is associated with a reduced risk of cancer [16–18]. As well as in the case of diabetes, the explicit mechanism of its antineoplastic action is not yet clear [11]. Metformin was proposed to act either indirectly by decreasing levels of insulin [19] or directly by suppression of mitochondrial-dependent biosynthetic pathways [20, 21]. One of the most studied possible molecular targets for biguanides action is their inhibitory action on respiratory chain complex I (NADH dehydrogenase, NDH), first described in liver tissue [8, 22]. Since then, it was confirmed for various tissues and cellular models and crucially also for cancer cells [11, 23, 24]. Hypothetical model proposes that NDH inhibition leads to decreased respiration and consequently to activation of AMPK, the key player in cellular energy homeostasis [25]. The exact site of biguanide binding to NDH is ambiguous; it was found to influence reactivity of its flavin cofactor, but it also inhibits ubiquinone reduction in a noncompetitive manner [23]. In addition to NDH, metformin was shown to inhibit other enzymes of the mitochondrial oxidative phosphorylation apparatus, including mitochondrial glycerophosphate dehydrogenase (mGPDH) and ATP synthase [7, 10, 23]. Interestingly, the viability of cancer cells lacking mitochondrial DNA (rho0 cells) is also affected by the drug, making its putative action on respiratory chain complexes rather questionable [26].

Respiratory chain is also a predominant source of reactive oxygen species (ROS), and targeting individual complexes may modulate ROS production and thus influence various pathological processes and/or signaling in different metabolic pathways [27]. Using specific substrates and inhibitors of respiratory chain enzymes, it is possible to localize the site of ROS generation or conversely the binding site of the inhibitor. In isolated mitochondria, the electron leak occurs mainly under the conditions of high NAD(P)H pool reduction [28, 29] or high ubiquinone pool reduction (high proton motive force) [29–31]. Essentially, all of the dehydrogenases in the respiratory chain were under certain conditions demonstrated to allow electron leak and ROS production. Flavin site of complex I (I_F_) was identified as a site of superoxide production using NADH-linked substrates [32, 33]. Under the conditions of high flux from succinate oxidation and high proton motive force, the electrons can backflow to NDH and escape at the level of Q site (I_Q_) towards the molecular oxygen [30, 31]. SDH itself was shown to produce significant amounts of ROS, especially when succinate levels are low (submillimolar). Under these conditions, flavin site (II_F_) is not fully occupied by the substrate and is more accessible to oxygen allowing superoxide generation [34]. Mitochondrial mGPDH was also shown to act as a potent ROS producer [35] even in mitochondria from tissues with low amount of the enzyme [36]. ROS production from mGPDH can reach the levels of ROS from complex III when inhibited with antimycin A (the most potent source of ROS in mitochondria) [37]. Since mGPDH-dependent ROS production increases linearly with increasing GP concentration, the most plausible site of electron leak is the Q site or the semiquinone formed here [27].

Given the conflicting reports regarding the molecular target of biguanides in mitochondria, in this study, we addressed the direct impact of biguanides metformin and phenformin on NDH, SDH, and mGPDH, mitochondrial glycerophosphate dehydrogenases, which feed electrons into respiratory chain and were proposed to be a target of these drugs. We used mitochondria isolated from brown adipose tissue (BAT), which are advantageous for such study as they contain comparable amounts of these dehydrogenases. Furthermore, uncoupling protein 1 (UCP1) dissipates mitochondrial membrane potential (Δ*ψ*_m_) in the isolated BAT mitochondria so that the cationic biguanides are much less concentrated in mitochondrial matrix, and therefore, the determined concentrations of these drugs are very close to the actual ones required for inhibition. We compared the effects of biguanides on dehydrogenases with their typical inhibitors (rotenone, atpenin A5, and iGP-1) using activity assays, mitochondrial oxygen consumption, and ROS production.

## 2. Materials and Methods

### 2.1. Isolation of Mitochondria from Brown Adipose Tissue

We used interscapular brown adipose tissue (BAT) of four-week-old Wistar rats kept at 22°C under 12 h/12 h light/dark cycle on a standard diet and water supply ad libitum. All animal works were approved by the institutional ethics committee and were in accordance with the EU Directive 2010/63/EU for animal experiments. Mitochondria were isolated in STE medium (250 mM sucrose, 10 mM Tris–HCl, 1 mM EDTA, pH 7.4) and supplemented with BSA (10 mg/mL) by differential centrifugation [38]. Quality of isolation was routinely checked by oxygraph, and fresh mitochondria were used for measurements of oxygen consumption and hydrogen peroxide production. Subsequently, frozen-thawed mitochondria were used for determination of enzymatic activities; as in such preparations, the integrity of mitochondrial membrane is disrupted and NADH has therefore access to the oxidation site of NDH. Also, freezing/thawing ensures that the membrane potential is not maintained.

### 2.2. Enzyme Activity Assays

Activities of mitochondrial dehydrogenases were determined spectrophotometrically as NADH oxidoreductases (NDH, monitored at 340 nm, *ε*_340_ = 6.22 mM^−1^·cm^−1^) or CoQ_1_ oxidoreductases (SDH and mGPDH, monitored at 275 nm, *ε*_275_ = 13.6 mM^−1^·cm^−1^). The assay medium contained 50 mM KCl, 20 mM Tris–HCl, 1 mM EDTA, 1 mg/mL BSA, 2 mM KCN, pH 7.4, and 50 *μ*M CoQ_1_. The reaction was started by adding 100 *μ*M NADH, 25 mM glycerophosphate (GP), or 25 mM succinate, respectively, and after 5–10 minutes, changes of absorbance were monitored at 30°C. Enzyme activities were expressed as pmol/s/mg protein.

### 2.3. Determination of Mitochondrial Membrane Potential

The changes in mitochondrial membrane potential (Δ*ψ*_m_) were measured with TPP^+^-selective electrode as described in [39, 40]. Isolated BAT mitochondria (0.4 mg/mL) were resuspended in KCl medium and subsequently supplemented with 10 mM GP, 0.3% BSA, 1 mM GDP, and 1 *μ*M carbonyl cyanide-p-trifluoromethoxyphenylhydrazone (FCCP). The electrode was calibrated by stepwise addition of TPP^+^ (1–6 *μ*M) before each measurement, and Δ*ψ*_m_ changes were plotted as pTPP, that is, the negative common logarithm of TPP^+^ concentration.

### 2.4. Fluorometric Detection of Hydrogen Peroxide Production

Hydrogen peroxide production was determined fluorometrically by measuring oxidation of Amplex UltraRed (Thermo Fisher) essentially as before [27]. Fluorescence of the Amplex UltraRed oxidation product was measured at 37°C using Tecan Infinite M200 multiwell fluorometer. Excitation/emission wavelengths were 544 nm (bandwidth 15 nm)/590 nm (bandwidth 30 nm). The assay was performed with 10 *μ*g of mitochondrial protein in KCl-based medium (120 mM KCl, 3 mM HEPES, 5 mM KH_2_PO_4_, 3 mM MgSO_4_, 1 mM EGTA, 3 mg/mL BSA, pH 7.2) supplemented either with 10 mM pyruvate plus 2 mM malate, 10 mM succinate, or 10 mM GP. Amplex UltraRed was used at the final concentration of 50 *μ*M with horseradish peroxidase (HRP) at 1 U/mL. Fluorescence signal from the well containing all substrates and inhibitors, but not mitochondria, was subtracted as a background for every experimental condition used. Background, caused mostly by autoxidation of the dye or nonenzymatic effect of inhibitors on apparent ROS production, varied between conditions from 0 to 1 pmol/s/mg and was uniform across respective titration points. Signal was calibrated using H_2_O_2_ at the final concentration of 0–5 *μ*M, and H_2_O_2_ stock concentration was routinely checked by measuring its absorption at 240 nm.

### 2.5. Western Blotting

BAT and liver homogenates were denatured at 65°C for 15 min in a sample lysis buffer (2% (*v*/*v*) 2-mercaptoethanol, 4% (*w*/*v*) SDS, 50 mM Tris–HCl, pH 7.0, 10% (*v*/*v*) glycerol, and 0.017% (*w*/*v*) Coomassie Brilliant Blue R-250), and Tricine SDS-PAGE [41] was performed on 10% (*w*/*v*) polyacrylamide slab gels at room temperature. The gels were blotted onto a PVDF membrane (Immobilon P, Merck Millipore) by semidry electrotransfer at 0.8 mA/cm^2^ for 1 h. Membranes were blocked in 5% nonfat dried milk dissolved in TBS (150 mM NaCl, 10 mM Tris–HCl, pH 7.5) for 1 h at room temperature and incubated for 2 h with the following primary antibodies: antibody to NADH dehydrogenase (NDUFA9 subunit of complex I)—Abcam ab14713, succinate dehydrogenase complex (subunit A (SDHA) of complex II)—Abcam ab14715, Core2 subunit of complex III—Abcam ab14745, actin—Millipore MAB1501, rabbit polyclonal antibody to porin (1 : 1000) was a kind gift from Professor Vito de Pinto (Dipartimento di Scienze Chimiche, Catania, Italy), and rabbit polyclonal antibody to mGPDH was custom prepared [42]. Membranes were then incubated for 1 h with corresponding secondary fluorescent antibodies, IRDye 680- or 800-conjugated donkey anti-mouse IgG (Thermo Fischer) or donkey anti-rabbit IgG (LI-COR Biosciences), respectively. The fluorescence was detected using ODYSSEY infra-red imaging system (LI-COR Biosciences), and the signal was quantified using Aida 3.21 Image Analyzer software (RayTest).

### 2.6. Polarographic Detection of Oxygen Consumption

Oxygen consumption was measured at 30°C as described before [35] using Oxygraph-2k (Oroboros, Austria). Measurements were performed in 2 mL of KCl medium (80 mM KCl, 10 mM Tris–HCl, 3 mM MgCl_2_, 1 mM EDTA, 5 mM K-Pi, pH 7.4) using 15–60 *μ*g protein/mL of freshly isolated mitochondria. For measurements, 10 mM pyruvate plus 2 mM malate, 10 mM succinate, or 10 mM GP, respectively, were used. The oxygen consumption was expressed in pmol oxygen/s/mg protein.

## 3. Results and Discussion

The direct mechanistic knowledge on how biguanides (metformin, phenformin) influence mitochondrial function is not yet clear. While their inhibitory effect on NADH dehydrogenase (complex I, NDH) received most attention [8, 23, 24], it was also reported that biguanides can inhibit other dehydrogenases in the mitochondrial respiratory chain, namely, succinate dehydrogenase (SDH) [10] and mitochondrial glycerophosphate dehydrogenase (mGPDH) [7]. As those dehydrogenases substantially differ in their architecture of substrate sites, coenzyme Q (CoQ), binding sites and pathways of the electron transfer from substrate to CoQ, such as lack of selectivity, is rather surprising. Therefore, we focused on the action of biguanides on NDH, SDH, and mGPDH and compared it with canonical specific inhibitors of ubiquinone binding site of complex I (rotenone) [31], ubiquinone site of SDH (atpenin A5) [43], and the novel specific inhibitor of mGPDH (iGP-1) [44].

**Figure 1:**
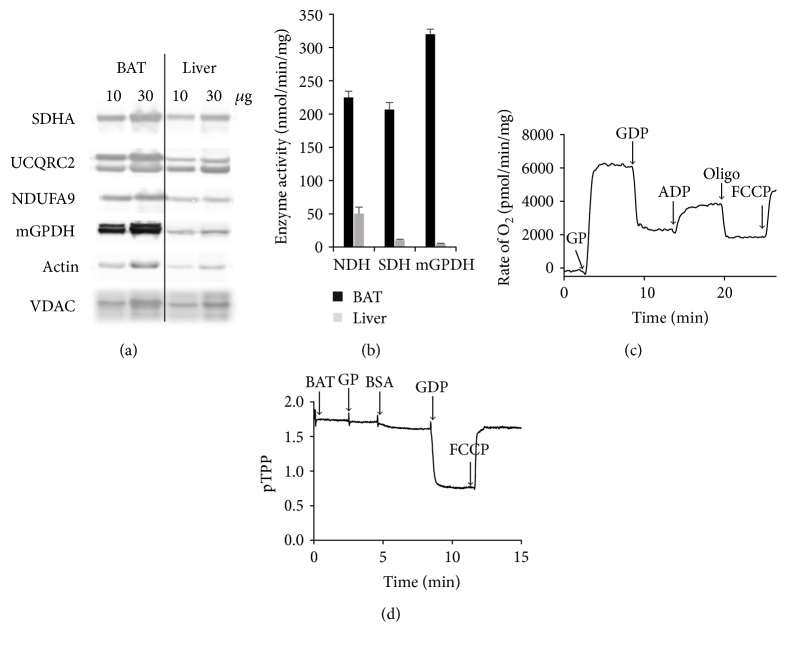
Characterization of brown adipose tissue (BAT) mitochondria. (a) SDS-PAGE and Western blot analysis of mitochondrial respiratory chain complexes in BAT and liver homogenates by polyclonal antibodies against mGPDH and VDAC and monoclonal antibodies against representative subunits of NDH (subunit NDUFA9), SDH (subunit SDHA), complex III (subunit UCQRC2), and actin. Two protein concentrations were loaded as indicated, representative image of three biological replicates. (b) Enzyme activities of mitochondrial dehydrogenases in mitochondria isolated from the BAT and liver. Activities were determined as rates of CoQ_1_ reduction using respective substrates. Results are means ± SEM from three independent measurements. (c) Representative quality control curve of O_2_ consumption and (d) representative trace of Δ*ψ*_m_ measurement in isolated BAT mitochondria. The following compounds were added: 10 mM glycerophosphate (GP), 0.3% BSA, 1 mM GDP, 1 mM ADP, 1 *μ*M oligomycin (oligo), and 1 *μ*M carbonyl cyanide-p-trifluoromethoxyphenylhydrazone (FCCP).

As a model, we chose isolated mitochondria from brown adipose tissue (BAT), as they have several advantages for such study. First, compared to the liver, where the inhibitory action of biguanides on mGPDH was originally described [7], they contain near equimolar levels of all three dehydrogenases studied. This can be documented both at the level of protein quantity (Figure 1(a)) and enzyme activity (NDH, SDH, and mGPDH, Figure 1(b)). The differences in response between dehydrogenases in BAT cannot therefore be ascribed to the varying content of enzymes studied. It is well established [36] and also obvious from Figure 1(b) that mGPDH activity in the liver is particularly low, and this can render the accurate measurement of inhibitory effects on GQR quite difficult. Another advantage of BAT mitochondria is the presence of UCP1 protein [45] which under native conditions allows the flow of protons back to the matrix (Figure 1(c)) and thus effectively discharges the mitochondrial membrane potential to the same level as prototypic uncoupler FCCP as can be seen by direct Δ*ψ*_m_ determination on TPP^+^-selective electrode (Figure 1(d)). This means that studied ROS production is independent of OXPHOS coupling. On the other hand, mitochondria can easily be coupled by the inhibitory action of guanosine diphosphate (GDP) on the UCP1 and contribution of electron backflow can then be established. Finally, lack of coupling together with physiologically low levels of mitochondrial F_o_F_1_ ATP synthase makes the action of biguanides on mitochondrial dehydrogenases rather independent of ATP synthase activity. This is quite important as F_o_F_1_ ATP synthase was also shown to be inhibited by biguanides [23] and thus may blur the picture in other model systems.

### 3.1. Effect of Biguanides on Enzymatic Activities

**Figure 2:**
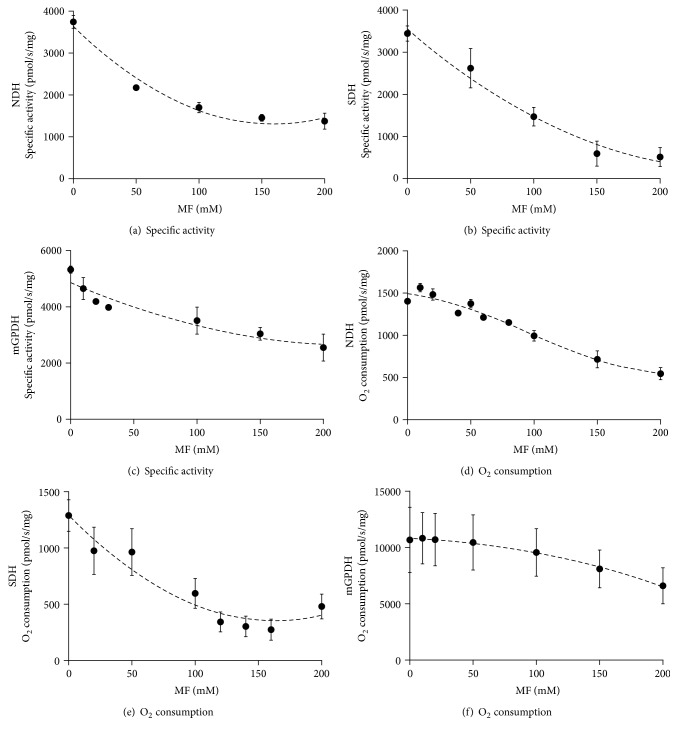
Respiratory chain dehydrogenase sensitivity to metformin titration. Specific enzyme activities (a, b, c) or oxygen consumption (d, e, f) of BAT mitochondria was titrated with 0–200 mM metformin using 100 *μ*M NADH (a) or 10 mM pyruvate plus 2 mM malate (d), 10 mM succinate (b, e), and 25 mM (c) or 10 mM glycerophosphate (f). Individual points represent means ± SEM of at least three independent measurements.

**Figure 3:**
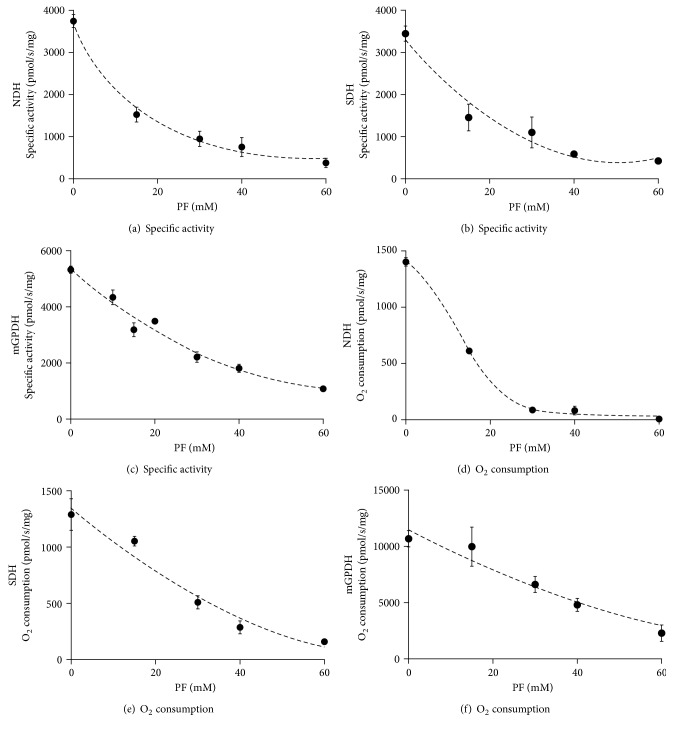
Respiratory chain dehydrogenases sensitivity to phenformin titration. Specific enzyme activities (a, b, c) or oxygen consumption (d, e, f) of BAT mitochondria was titrated with 0–60 mM phenformin using 100 *μ*M NADH (a) or 10 mM pyruvate plus 2 mM malate (d), 10 mM succinate (b, e), and 25 mM (c) or 10 mM glycerophosphate (f). Individual points represent means ± SEM of at least three independent measurements.

As a first step, we estimated direct impact of metformin on activity of each complex measured as individual dehydrogenase activity (substrate : coenzyme Q) in frozen-thawed mitochondria or in the context of electron transport chain in intact mitochondria measured as oxygen consumption using respective substrates (Figure 2). Metformin inhibited all enzymes studied, but only at very high concentrations (IC_50_ varied from 80 to 180 mM, Figures 2(a), 2(b), and 2(c)). Out of those activities, NDH was the most sensitive to the inhibitory action of metformin (Figure 2(a)). Similarly, at the same concentrations, metformin also inhibited respiration with pyruvate and malate used as substrates for NDH (Figure 2(d)), succinate for SDH (Figure 2(e)), and glycerophosphate (GP) for mGPDH (Figure 2(f)). Another biguanide, phenformin, behaved analogously to metformin, but its inhibitory effect was more efficient; the IC_50_ varied between 10 and 25 mM for both isolated enzyme activities and respiration of all three dehydrogenases (Figure 3). Because of the limited permeability of the biguanides, we waited to reach plateau (approximately 5 min) before proceeding to the next addition. It is to be stressed that IC_50_ values of observed inhibitory effects in uncoupled mitochondria (oxygraphy) were the same or slightly higher than corresponding IC_50_ values in frozen-thawed mitochondria (spectrophotometry), thus conferring comparable concentration of biguanides on both sides of the inner mitochondrial membrane. Determined IC_50_ in our case is rather high in comparison with that previously reported [7, 33]. This can be most likely attributed to the uncoupled state of BAT mitochondria which prevents accumulation of biguanides in mitochondria (see below).

**Figure 4:**
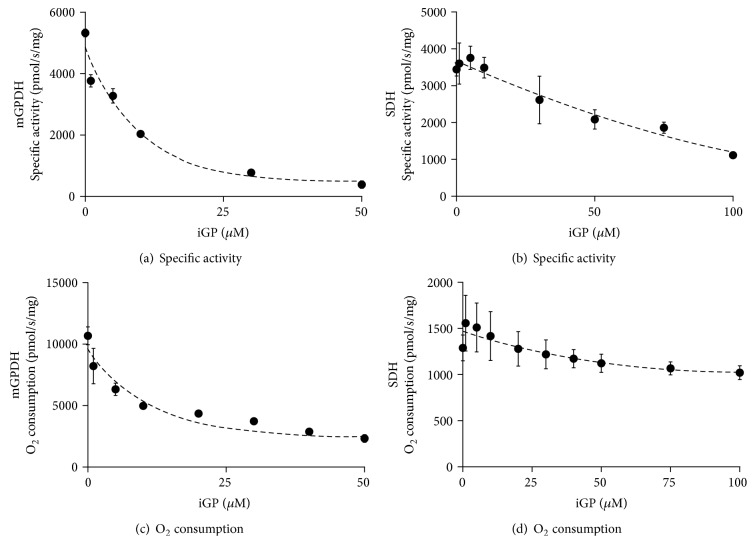
Comparison of iGP-1 effect on respiratory chain dehydrogenases mGPDH and SDH. Specific enzyme activities (a, b) or oxygen consumption (c, d) of BAT mitochondria was titrated with 0–100 *μ*M iGP-1 using 25 mM (a) or 10 mM glycerophosphate (c) and 10 mM succinate (b, d). Individual points represent means ± SEM of at least three independent measurements.

Subsequently, we focused on the action of canonical inhibitors on individual complexes. As expected, rotenone completely inhibited complex I enzyme activity as well as pyruvate plus malate-supported oxygen consumption at 500 nM. Similarly, complete inhibition of SDH by atpenin A5 using succinate as a substrate was achieved at even lower concentrations (20 nM) and activity of mGPDH was abolished at 50 *μ*M of iGP-1. Interestingly, we also observed some nonspecific actions of iGP-1 on SDH (Figures 4(b) and 4(d)), but the IC_50_ was significantly higher (~80 *μ*M) than that in the case of mGPDH (Figures 4(a) and 4(c)).

Given the extremely high IC_50_ we identified for phenformin and metformin, it is to be questioned whether their effects on respiratory chain enzymes are pharmacologically relevant and can affect mitochondrial function in tumors. As already mentioned, isolated BAT mitochondria with inherently low levels of mitochondrial membrane potential represent a model free of the effect of metformin partitioning due to its charge. Biguanides carry a positive charge, which means that they do accumulate in cells and mitochondria in dependence on membrane potential across both cellular and mitochondrial membranes. Thus, the concentration could be 100 to 300 times higher inside energized mitochondria (compared to concentration in cytosol) with the difference in mitochondrial membrane potential between 120 and 150 mV [23]. Since the reported values for metformin concentration inside tumors are in 1–10 *μ*M range [21], the actual concentration in respiring mitochondria could reach mM values, which is likely not sufficient for the inhibitory action on mitochondrial oxidative phosphorylation. Indeed, in this regard, it is important to note that two recent reports demonstrated higher tumor antiproliferative efficiency for mitochondrially targeted (through positive charge of conjugated TPP moiety) analogs of metformin [46, 47]. However, metformin and its conjugated analogs accumulate in the mitochondrial matrix, where they can act on enzyme complexes facing this compartment, namely, NDH and SDH. The effect on mGPDH is more questionable, since this enzyme is located on the outer face of the inner membrane, facing the mitochondrial intermembrane space and does not span to the matrix [48]. One of the reports using mitochondrially targeted metformin analogs [46] reported also higher potency of that compound on GP-dependent respiration, which would be in line with rather generalized nonspecific mode of action for example through the influencing of mitochondrial membrane properties.

### 3.2. Reactive Oxygen Species Production

Action of pharmacologically active compounds on suppression of cancer cell proliferation may not only be achieved by decreased flow of electrons through respiratory chain and associated impairment of aerobic ATP production. Another successful antiproliferative strategy may be to increase in reactive oxygen species (ROS) production and subsequent induction of apoptosis [49, 50]. It was also proposed for biguanides that their primary antiproliferative effect is manifested through increase in ROS production [21, 23], which also holds true for the mitochondria-targeted analogs [51]. In this case, the mitochondrial ROS also interfered with redox signaling events. Mitochondrial respiratory chain contains numerous sites which may leak electrons to molecular oxygen and produce superoxide [27, 34, 35, 52–55]. The major superoxide-producing sites in mitochondrial respiratory chain were proposed to be NDH [53, 56], complex III [53, 57], mGPDH [27, 35], and, under certain circumstances, also SDH [34].

**Figure 5:**
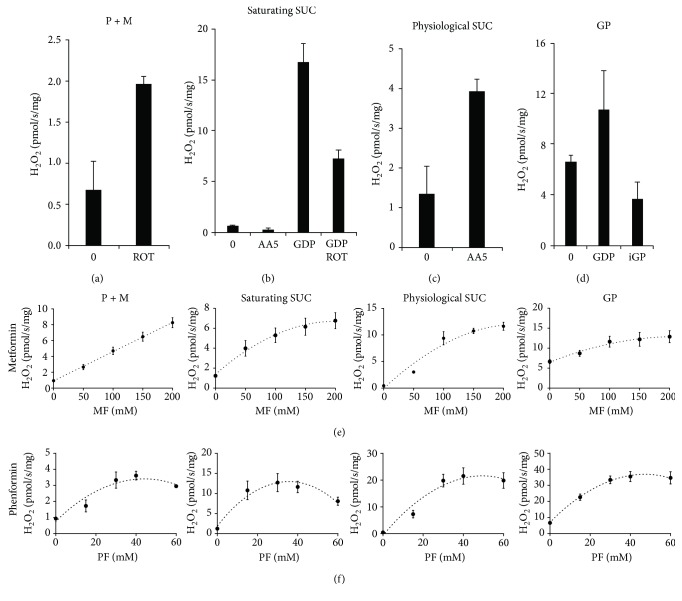
Effect of biguanides on reactive oxygen species (ROS) production compared to typical inhibitors. The rate of H_2_O_2_ generation was estimated by Amplex red assay. H_2_O_2_ production with (a) 10 mM pyruvate and 2 mM malate (P + M) with or without 1 *μ*M rotenone (ROT), (b) 10 mM succinate (saturating SUC) with or without 0.5 *μ*M atpenin A5 (AA5) or 1 *μ*M rotenone, (c) 0.4 mM succinate (physiological SUC) with or without 0.5 *μ*M atpenin A5, and (d) 10 mM glycerophosphate (GP) with or without 100 *μ*M iGP-1 (iGP) were indicated; BAT mitochondria were coupled with 1 mM guanosine diphosphate (GDP). (e) Titration of H_2_O_2_ production by biguanides metformin (MF, 0–200 mM) or (f) phenformin (PF, 0–60 mM) using the same substrate concentrations as in respective experiments in (a–d). Each point is the mean ± SEM of at least three independent measurements.

To distinguish sites in mitochondrial respiratory chain, where biguanides may induce electron leak, we followed the rates of ROS production (detected as H_2_O_2_ upon conversion by superoxide dismutase) in fresh mitochondria from BAT, where the respiratory chain is not reduced due to uncoupling by UCP1. First, using the established inhibitors of individual dehydrogenases and their respective substrates, we established ROS production pattern characteristic for each site in BAT mitochondria (Figure 5). As expected, also in BAT mitochondria, rotenone increased the rate of H_2_O_2_ production with NADH-linked substrates (pyruvate and malate), indicative of ROS production from I_F_ site (Figure 5(a)).

Using high concentrations of succinate (10 mM), very low levels of ROS were produced in uncoupled state, which is in agreement with the previously published data [29] and this leak occurs at the Q site of SDH (Figure 5(b)). After GDP coupling, ROS were produced by the electron backflow at the I_Q_ site of NDH (Figure 5(b)). Low levels of succinate led to pronounced increase in ROS production from flavin of SDH (inhibition of the Q site by atpenin A5, Figure 5(c)).

GP-dependent ROS production was quite high even when respiratory chain was not reduced and did not increase further after GDP addition, again in agreement with previously reported data [37, 58], and it was inhibited by mGPDH inhibitor iGP-1 (Figure 5(d)).

Compared to typical inhibitors, both metformin and phenformin induced dose-dependent increase in ROS production, but it seemed rather indiscriminative, as its pattern was highly similar for all substrates tested (Figures 5(e) and 5(f)). It suggests that these compounds do not bind directly to either flavin or ubiquinone-binding site of either of the dehydrogenases studied and that biguanides can affect mitochondria by different mechanisms, for example, they can influence membrane phospholipid environment.

## 4. Conclusions

**Figure 6:**
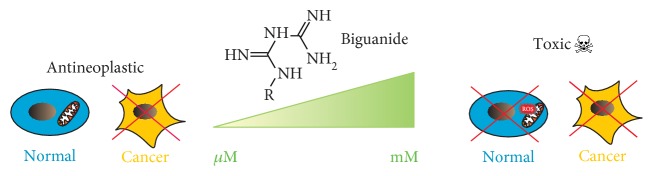
Proposed scheme of biguanide action. While at micromolar concentrations, biguanides confer antineoplastic action; at millimolar ones, they inhibit mitochondrial respiratory chain complexes and are toxic for all the cells.

Our data demonstrate that the inhibitory effect of biguanides on OXPHOS enzymes is rather pleiotropic including the previously reported inhibition of NADH and mGPDH dehydrogenases. Drug sensitivity of respiratory chain complexes in brown adipose tissue was comparable, with IC_50_ higher than 80 mM in case of metformin or ranging from 20 to 30 mM in case of the more potent phenformin. Moreover, these biguanide concentrations induce nonspecific increase of mitochondrial reactive oxygen species production. Our data suggest that biguanides do not bind to any specific sites on respiratory chain dehydrogenases and require high concentrations to be effective. Under these conditions, their effect on dehydrogenases remains therapeutically questionable. Indeed, phenformin use had to be discontinued due to induction of lactic acidosis in treated patients. As summarized in Figure 6, we propose that pharmacologically relevant concentrations of biguanides confer their antineoplastic effect through yet unidentified target in mitochondrial metabolism, different from the inhibition of individual dehydrogenases.

## Abbreviations

BAT: Brown adipose tissue
NDH: NADH dehydrogenase
SDH: Succinate dehydrogenase
mGPDH: Mitochondrial glycerophosphate dehydrogenase
OXPHOS: Oxidative phosphorylation
GDP: Guanosine diphosphate
ROS: Reactive oxygen species
MF: Metformin
PF: Phenformin
UCP1: Uncoupling protein 1
I_Q_: Coenzyme Q binding site of respiratory chain complex I
I_F_: Flavin site of respiratory chain complex I
II_F_: Flavin site of respiratory chain complex II.
